# Non-enzymatic disposable electrochemical sensors based on CuO/Co_3_O_4_@MWCNTs nanocomposite modified screen-printed electrode for the direct determination of urea

**DOI:** 10.1038/s41598-023-28930-4

**Published:** 2023-02-04

**Authors:** Hend S. Magar, Rabeay Y. A. Hassan, Mohammed Nooredeen Abbas

**Affiliations:** 1 Applied Organic Chemistry Department, National Research Centre, P.O. Box. 12622, Dokki, Cairo Egypt; 2 Nanoscience Program, University of Science and Technology (UST), Zewail City of Science and Technology, Giza, 12578 Egypt

**Keywords:** Biochemistry, Chemistry, Materials science, Nanoscience and technology

## Abstract

A new electrochemical impedimetric sensor for direct detection of urea was designed and fabricated using nanostructured screen-printed electrodes (SPEs) modified with CuO/Co_3_O_4_ @MWCNTs. A facile and simple hydrothermal method was achieved for the chemical synthesis of the CuO/Co_3_O_4_ nanocomposite followed by the integration of MWCNTs to be the final platform of the urea sensor. A full physical and chemical characterization for the prepared nanomaterials were performed including Fourier-transform infrared spectroscopy (FTIR), Raman spectroscopy, X-ray diffraction (XRD), X-ray photoelectron spectroscopy (XPS), contact angle, scanning electron microscope (SEM) and transmission electron microscopy (TEM). Additionally, cyclic voltammetry (CV) and electrochemical impedance spectroscopy (EIS) were used to study the electrochemical properties the modified electrodes with the nanomaterials at different composition ratios of the CuO/Co_3_O_4_ or MWCNTs. The impedimetric measurements were optimized to reach a picomolar sensitivity and high selectivity for urea detection. From the calibration curve, the linear concentration range of 10^−12^–10^−2^ M was obtained with the regression coefficient (R^2^) of 0.9961 and lower detection limit of 0.223 pM (S/N = 5). The proposed sensor has been used for urea analysis in real samples. Thus, the newly developed non-enzymatic sensor represents a considerable advancement in the field for urea detection, owing to the simplicity, portability, and low cost-sensor fabrication.

## Introduction

Owing to an imbalance of blood substances, various diseases such as cardiovascular, diabetes, cancer, kidney, heart disease, liver disorders, tuberculosis and chronic respiratory diseases are produced. It is important to decrease ingredients of blood such as uric acid, cholesterol, glucose and urea for human health^1,2^. Urea is produced in the liver, and moved to the kidneys by the bloodstream and extracted by the urine, where the protein metabolism is the end product. Normal level of urea in the human serum is ranging from 15 to 40 mg/dl and more than this permissible level can cause a critical diseases such as ulcers, acidity, digestion, urinary tract obstruction, renal failure, malfunctioning of kidneys, cancer, burns, shock, dehydration, gastrointestinal bleeding, and other health complications. However, less than the normal state can cause cachexia, hepatic nephritic syndrome and failure^3^.

Urea is normally found in different fields including dairy, agriculture, food preservation, fishery and its detection is necessary. In milk, urea testing is very important; the normal concentration of urea is 18–40 mg/dl^4^. In the pharmaceutical field, urea is found in many ointment products as a component and its level must be controlled. In groundwater or rivers, urea levels give indication of sewage contamination. Furthermore, the high level of urea is one of the reasons for algae blooming^5^. Thus, it is necessary to provide sensitive detection methods that can track the urea level with high sensitivity and selectivity^6–8^. To that end, conventional analytical techniques such as infrared (IR) spectrometry^9,10^, liquid chromatography (HPLC)^11^, NNMR^12^, calorimetry^13^, gas chromatography (GC)^14^, fluorimetry^15^ and chemiluminescence have been exploited for urea determination in real samples. However, these methods are complicated, require sample pretreatment, and long-time for analysis, in addition to the high interfering levels with other species such as K^+^, Na^+^ ions, uric acid^16,17^.

Alternatively, electrochemical biosensors provided low cost, rapid detection and high selectivity^18,19^^.^ Biosensors are bendable detection techniques that have high importance, being able to resolve a potential number of problems and industrial challenges in diverse areas such as defense-related issues, explosives, food safety, environmental monitoring^20–22^, drugs and pharmaceutical analysis^23^, disease biomarkers^24,25^, homeland safety^26,27^. In terms of sampling conditions, using disposable biosensor chips, target analytes could be monitored directly in a variety of complex samples without any prior sample pre-treatments^19,28,29^. Moreover, simultaneous analysis for multi-targets could be achieved with high reduction of costs and sample size, full-automation, selective identification and accurate recognition^30^.

Among the electrochemical biosensor techniques, electrochemical impedance spectroscopy (EIS) biosensors is the most powerful tools used for studying the bio-recognition events such drug-target identification, protein–protein affinity binding, or the antigen–antibody interaction^18,31,32^.

To design and fabricate an effective sensing platform, working electrode materials must satisfy certain criteria such as the low cost of materials, high electro-catalytic activity, biocompatibility and electrical conductivity. Accordingly, different nanostructured materials (e.g. nano-pores, nanowires, nanotubes, etc.) have been intensively explored^33–35^. Metal oxide nanostructures have unique electrochemical, catalytic and electronic properties that are controlled by their shape, size, and surface morphology^36–40^. Transition metal oxides (ZnO, NiO, MnO_2_, or CuO) have been used in enzymatic (urease enzyme) sensor for urea detection^41^, these nanostructures had a poor performance and not used for practical applications. On the other hand, NiO nanostructures have been used for non-enzymatic urea sensors with a poor conductivity, narrow range concentration and high detection limit of urea^42^. On the other hand, carbon-based materials, such as graphene or carbon nanotubes (single walled carbon nanotubes (SWCNTs) or multi-walled carbon nanotubes (MWCNTs), have been exploited to enable direct bioelectrochemical detection of many targets including small molecules^43–46^ and pathogenic or nonpathogenic microorganisms^33,34,47–50^^.^

To that end, exploring a new class of nanostructure-based sensors is very important. Formation of hybrids of metal oxide nanostructures can be helpful for solving the problem of the slow charge transfer processes and poor electrical conductivity. As a common hybrid metal oxides, CuO/Co_3_O_4_ exhibited higher electron conductivity than the individual constituent (i.e. the Co_3_O_4_ or CuO). Therefore, this promising hybrid nanostructure has been used in various applications^51–53^. However, in the literature, there is no report for CuO/Co_3_O_4_ based non-enzymatic sensor for urea detection, which deserves to be used due to the outstanding electronic and physical properties.

In this work, a non-enzymatic impedimetric biosensor was designed and characterized; the analytic method was optimized, and applied for the fast urea detection. For this approach, disposable screen-printed electrodes were functionalized with a hybrid of metal oxide nanostructure consisting of CuO/Co_3_O_4_ integrated MWCNTs to provide the high electrochemical performance for the newly developed sensor.

## Materials and methods

### Reagents and materials

Cobalt nitrate [Co(NO_3_)_2_·6H_2_O)], multi-walled carbon nanotubes (MWCNTs), copper(II) nitrate [Cu(NO_3_)_2_.3H_2_O)], ammonium hydroxide (NH_4_OH), potassium hydroxide (KOH), potassium ferricyanide , potassium ferrocyanide , potassium chloride and urea were obtained from Sigma Aldrich. Sodium acetate and acetic acid were obtained from Fluka.

### Apparatus

CH-Instruments Inc. (CHI-660D) electrochemical workstation was used for the cyclic voltammetric (CV) and electrochemical impedance spectra (EIS) measurements. Screen printed electrodes (SPEs) (three-electrode-SPE chip that consisted of gold as the working, counter, and reference electrode) were obtained from Metrohm DropSens and have been used as the sensing platform. Fourier Transform Infrared (FTIR) spectra were produced by FT-IR-6100 from JASCO with scans from 4000 cm^−1^ up to 400 cm^−1^. X-ray diffraction (XRD) peaks are produced using Cu-Kα radiation (wavelength = 1.5418 Å) with 2θ range from 4.015 to 79.961.Raman spectroscopy measurements for the metal oxides nano-hybrid, MWCTS, and the nanocomposite were conducted at room temperature using Raman Spectroscopy (Confocal Raman microscope, WITech, alpha-300R, excitation laser 532 nm and Laser power 1mW) has been used for the nanomaterials characterizations. Each spectrum was recorded in the range of 100–4000 cm^−1^. Elemental chemical states of the nanocomposite (CuO/Co_3_O_4_@MWCNTs) were determined by the X-ray photoelectron spectroscopy (XPS) with AlKα radiation (A Thermo Scientific K-ALPHA spectrometer). Contact Angles of water were used for different samples wetting properties by using OCA 15EC (model produced by company of Data Physics Instrument Gmbh). Images of scanning electron microscope (SEM) and energy dispersive X-ray spectroscopy (EDX) were performed using JEOL-JSM-6390LV. Transmission electron microscope (TEM) images were produced by a JEOL JEM-1230.

### Synthesis of CuO/Co_3_O_4_ nanocomposite

A mixture of Cu(NO_3_)_2_·3H_2_O and Co(NO_3_)_2_.6H_2_O (2.0 mM of each metal salt) was prepared in deionized (DI) water. 20 ml of ammonia solution was added to the mixture of metal salt with stirring for 12 h. Afterwards, the solution was added in an autoclave reactor (Teflon-lined stainless steel) and heated at 200 °C for 10 h, then calcined at 600 °C for 6 h. In the last step, the mixture was filtered and washed by pure ethanol^42^. CuO, Co_3_O_4_ and the commercial MWCNTs nanostructures were characterized by using IR, Raman, XPS,XRD, contact angle, SEM and TEM. In FTIR spectra, spectrum was performed in the range of 400 to 4,000 cm^−1^. In contact angle measurement, 1.0 μl of deionized water was dropping on the surface of MWCNTs, CuO, Co_3_O_4_, CuO/Co_3_O_4_ and CuO/Co_3_O_4_@MWCNTs with a rate of 1.0 μl/s. Behaviors of water droplets on the surfaces coated with the nanomaterials were imaged by a digital camera. For the electrochemical characterization of metal oxide nano-hybrid, different ratios of CuO/Co_3_O_4_ nanocomposite (0:100, 20:80, 40:60, 60:40, 80:20, and 100:0%) were prepared via mixing and sonication the suspension for 2 h. From each homogenous metal oxide nano-hybrid, a thin film was formed onto the surface of the screen-printed electrode via drop casting before testing the electrochemical properties. For CuO/Co_3_O_4_@MWCNTs nanocomposite, different percentages of MWCNTs (0, 0.1, 0.3, 0.5, 0.7, 0.9, and 1.0%) were added to the best ratio of CuO/Co_3_O_4_ nano-hybrid by sonication for 2 h.

### Electrode modification with CuO/Co_3_O_4_@MWCNTs nanocomposite

Suspended solutions containing different percentages of CuO:Co_3_O_4_ metal oxides (MOs) nano-hybrid were sonicated for 1.0 h to reach a homogeneous aqueous dispersion. Then, different percentages of MWCNTs were mixed with the CuO/Co_3_O_4_ metal oxides nanocomposite structure and sonicated for 1.0 h. On the screen-printed electrodes (SPEs) working surface area, 5.0 μl of different nanostructures were drop-casted, and left to dry then electrochemical characterization (CV and EIS) was performed.

### Electrochemical measurements

**Figure 1:**
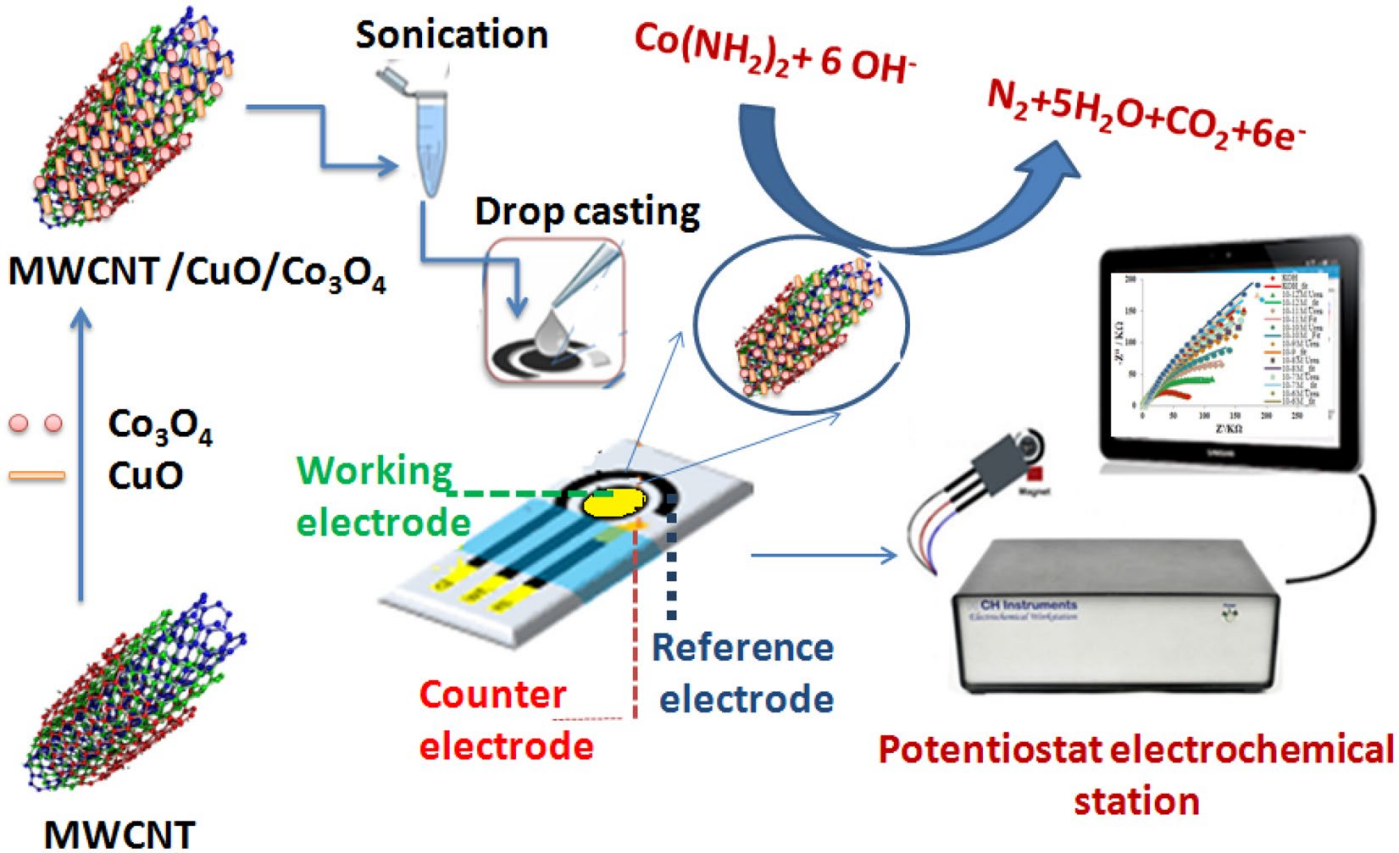
Schematic diagram showing the steps of fabrication of newly designed non-enzymatic urea sensor.

All electrochemical experiments were carried out by CHI-instrument at room temperature. EIS measurements were accomplished using three-electrode-SPE chip that consisted of gold as the working, counter, and reference electrode. The impedimetric measurements of different concentrations of urea were conducted in a solution of 0.1 M of KOH as an electrolyte, the range of frequency between 0.1 to 10^5^ Hz was applied at a potential of 0.7 V and with amplitude of 5.0 mV sinusoidal modulation. The electrochemical studies (CV and EIS) were measured in a solution of 0.1 M KCl and 5 mM of [Fe (CN)_6_]^3−/4−^ as a redox probe. CV measurements were conducted at the scan rate of 50 mV/s and range of potential from − 0.4 to + 0.7 V. For the quantitative EIS data analysis, an equivalent circuit model was simulated and used for fitting the nyquist impedance spectra. Sensor fabrication steps and SPEs functionalization with the nanomaterials along with the impedimetric measurements are presented in Fig. 1.

## Results and discussion

### Structural and morphological characterization of the nanomaterials

The prepared nanomaterials including the CuO/Co_3_O_4_ and CuO/Co_3_O_4_@MWCNTs nanocomposite were fully characterized using FTIR, Raman spectroscopy, XRD, XPS, EDX, SEM, TEM, and contact angle.

**Figure 2:**
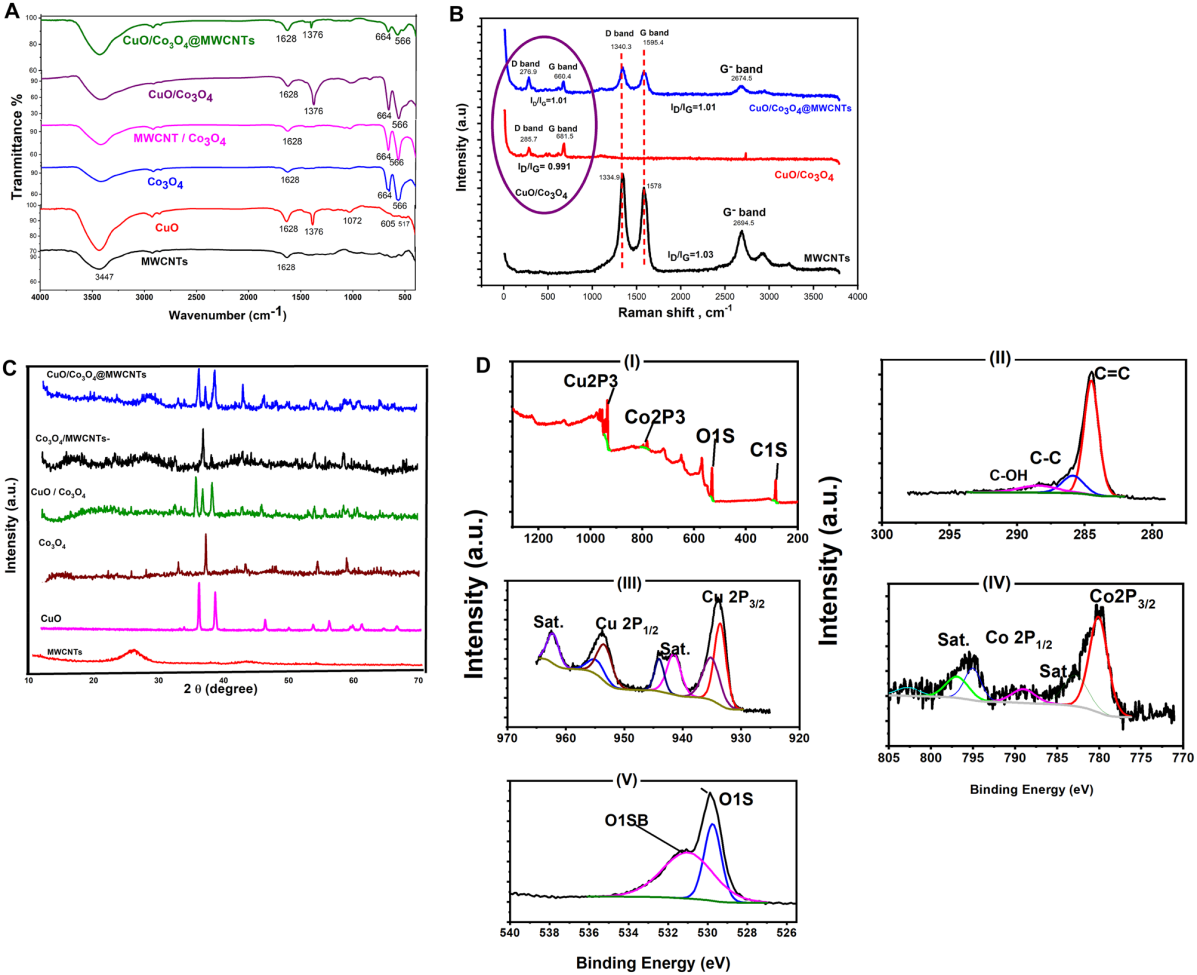
(**A**) FTIR spectrum of MWCNTs, CuO, Co_3_O_4_, Co_3_O_4_/MWCNTs, and CuO/Co_3_O_4_@MWCNTs. (**B**) Raman spectroscopy of MWCNTs, Co_3_O_4_, CuO, Co_3_O_4_/CuO, Co_3_O_4_/MWCNTs and CuO/Co_3_O_4_@MWCNTs nanocomposite. (**C**) Diffraction patterns (XRD) of MWCNTs, Co_3_O_4_, CuO, CuO/Co_3_O_4_, Co_3_O_4_/MWCNTs and CuO/Co_3_O_4_@MWCNTs nanocomposite. (**D**) X-ray photoelectron spectroscopy (XPS) spectra of as-fabricated CuO/Co_3_O_4_@MWCNTs electrode: (I) the survey spectrum, (II) C 1 s, (III) Cu 2p, (IV) Co 2p and (V) d O 1 s.

In the FTIR spectrum (see Fig. 2A) of MWCNTs, the absorption band of the O–H stretching group appeared at 3446 cm^−1^, while another band was obtained at 1627 cm^−1^ belonging to the C=O stretching group^54^. The obtained FTIR spectrum of CuO is correlated with the previously reported data, whereas three characteristic bands positioned at (517, 605, and 658 cm^−1^) were ascribed to Cu(II)-O stretching frequencies^55^. The sharp peak obtained at the 605 cm^−1^ in the CuO spectrum is referring to Cu–O bond formation. Additionally, a broad peak was observed at around 3420 cm^−1^ due to the adsorption of water molecules. On the other hand, Co_3_O_4_ FTIR spectrum demonstrated two essential stretching bands at 566 and 664 cm^−1^ reflecting the vibrational modes of the metal–oxygen (Co–O) in that spinel compound^56^.

To describe the probability of forming inter-forces between the metal oxide nano-hybrid and the MWCNTs in the formed nanocomposite, Raman spectroscopic analysis was performed. In this regard, the intensity ratio of the D and G bands (I_D_/I_G_) of each nanomaterial is used to demonstrate the binding forces change in the nanocomposite. As a result, a decrease in I_D_/I_G_ values from (1.03) of the MWCNTs to the I_D_/I_G_ value of (1.01) of the CuO/Co_3_O_4_@MWCNTs was obtained. This kind of change in the band intensity ratio was also combined with other changes that resulted from the metal oxides before and after their conjugation with the MWCNTs. As shown in Fig. 2B, the I_D_/I_G_ value of (0.99) was obtained from the metal oxides before they were mixed with the carbon nanotubes, while I_D_/I_G_ value of (1.01) was obtained after the combination was made. Furthermore, Raman shift of D and G band locations of the metal oxide nano-hybrid (285 and 781 cm^−1^) was observed which are higher than the location of the D and G bands of the nanocomposite that have been detected at 276 and 660 cm^−1^ , respectively. Eventually, Raman shifts in the location of the G' bands of the metals oxides and the nanocomposite was observed in the higher frequency side. Thus, from the changes obtained from the I_D_/I_G_ ratios, or from the Raman shifts, a strong interaction between the metal oxides nano-hybrid (CuO/Co_3_O_4_) and the MWCNTs could be concluded.

Further structural analysis was confirmed using the XRD analysis. The results (Fig. 2C) showed that there are various diffraction patterns associated with the CNTs, Co_3_O_4_ and CuO. For MWCNTs the diffraction peaks of 2θ obtained at 25.4, and 42.9 representing the crystal planes of (100), (002), respectively. For the diffraction pattern of Co_3_O_4_, the sharp peaks were appeared at 2θ values 21.07, 31.20, 37.05, 38.41, 45.03, 51.03, 60.23 and 66.10 that correspond to the crystal planes (111), (220), (311), (222), (400), (422), (511) and (440) respectively^57^. These results are strongly correlated with that obtained from the cubic phases of Co_3_O_4_ standard XRD data (similar to phase analysis ICSD card no. 98–006-3164). For the annealed monoclinic copper oxide at 600 °C, different diffraction peaks were detected at the 2θ values of 32.54, 35.51, 38.77, 48.74, 53.50, 58.34, 61.56, 65.83, 66.27,72.45 and 75.07 which are corresponding to the diffraction planes of (110), (002), (111), (-202), (202), (020), (-113), (-311), (220), (311) and (004), respectively. These findings were strongly matched with the reference XRD database of CuO (ICSD card no. 98–001-6025). Moreover, the annealed Co_3_O_4_/CuO at 600 °C showed the same diffraction peaks that obtained from Co_3_O_4_ and CuO. When the MWCNTs were added to the metal oxide nano-hybrid, no obvious changes in the crystallinity pattern were detected.

On the other hand, crystallite size of each of the presented nanomaterials was calculated from the giving Scherrer’s Eq. (1):

1 $$ {\text{D}} = 0.94\lambda /\beta \cos \theta \quad {\text{Scherrer}}^{\prime}{\text{s}}\;{\text{formula}} $$

where θ is the Bragg’s angle, λ is the X-ray wavelength, and β and θ are full widths at half maxima and Bragg’s angle of the XRD peak, respectively. On the other hand, the dislocation density (σ = 1/D^2^) of the nanomaterials was simply calculated from the obtained crystallite size values (Table S1, supplementary materials).

The elemental composition and valence state of the whole nanocomposite (CuO/Co_3_O_4_@MWCNTs) has been characterized by the X-ray photoelectron spectroscopy (XPS). In this regard, Fig. 2D-I demonstrated the complete XPS spectrum obtained for the CuO/Co_3_O_4_@MWCNTs nanocomposite powder, in which the elements of Cu, Co, O, and C are collected from the surface of the sample. The spectrum of MWCNTS (Fig. 2D-II) showed a majority of pure carbon (highest peak intensity at 284.5 eV) with minor traces of oxygen. Moreover, two main peaks at the binding energies of 933.5 and 953.8 eV attributed to the Cu2p_3/2_ and Cu2p_1/2_, respectively, and the two peaks with binding energies of 963.1 and 942.6 eV were assigned to the satellite peaks related to the corresponding main peaks (Fig. 2D-III), confirming the formation of Cu^2+^^58^^.^

In Fig. 2D-IV, two prominent peaks at the binding energies of about 779.6 and 795.6 eV referring to the Co2p_1/2_ and Co2p_3/2_, exhibiting the coexisting of both Co^2+^ and Co^3+^ in the sample and two satellite peaks in the spectrum confirming the presence of a dominant Co_3_O_4_ phase. Eventually, Fig. 2D-V, two peaks at the binding energy of 529.6, and 531.4 eV were obtained for the O1s to indicate the presence of the typical metal–oxygen bond. Collecting all given information in Fig. 2D, the successful formation of CuO/Co_3_O_4_@MWCNTs nanocomposite is confirmed.

**Figure 3:**
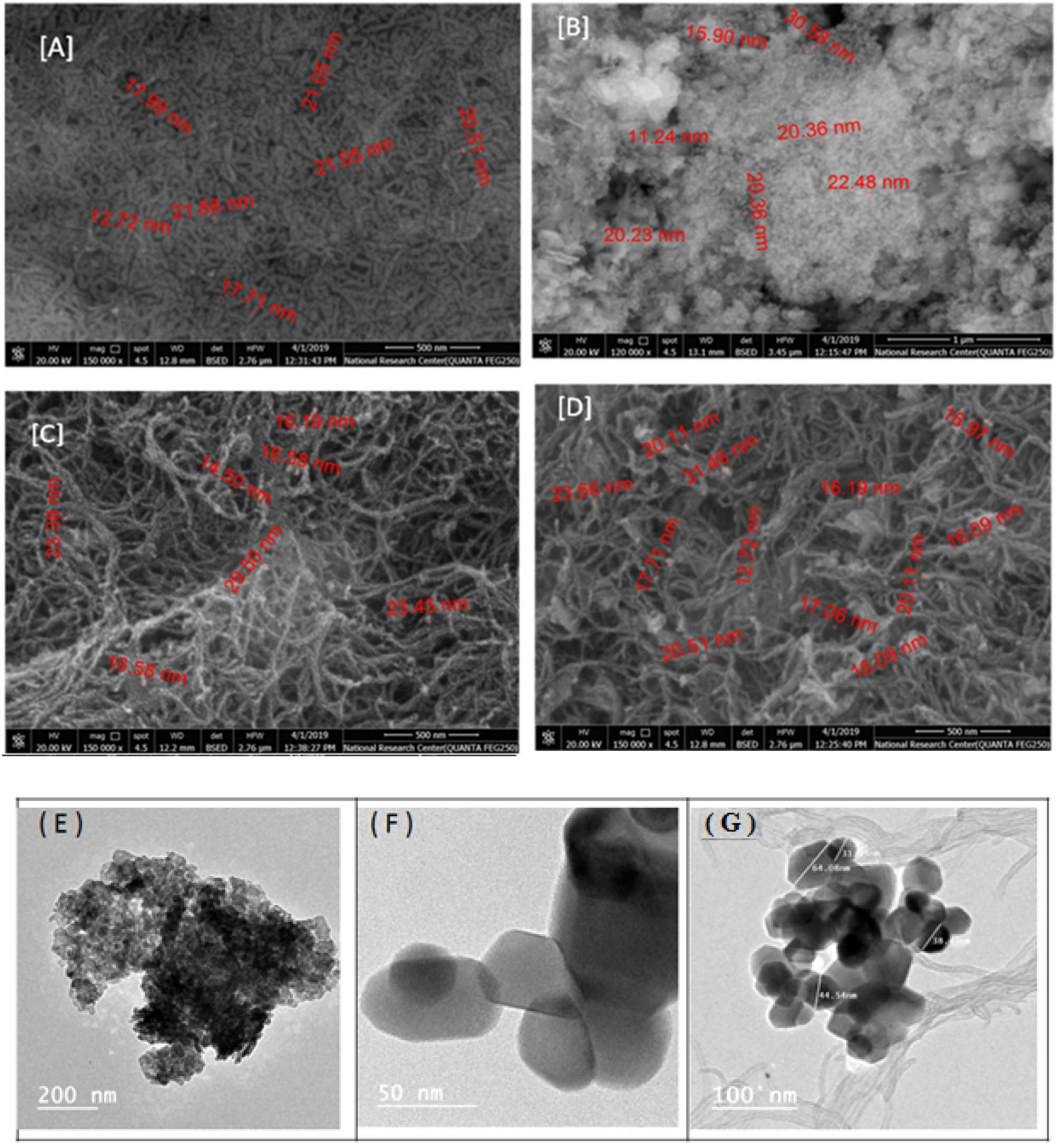
Morphological analysis using scanning electron microscopy (SEM) showing the 3D images of the (**A**) CuO, (**B**) CuO/Co_3_O_4_, (**C**) Co_3_O_4_/MWCNTs, and (**D**) CuO/Co_3_O_4_@MWCNTs. TEM images of (**E**) Co_3_O_4_, (**F**) CuO/Co_3_O_4_ and (**G**) CuO/Co_3_O_4_@MWCNTs.

For the topographical and morphological analysis, SEM and TEM images has been conducted on the nanocomposite and its individual constituents, as shown in Fig. 3. To that end, the 3D SEM images shown in Fig. 3 A&B demonstrated that the homogeneous formation of CuO, and Co_3_O_4_ nanoparticles with the average nano-sizes ranged from 30–40 nm. When the MWCNTs was added either to Co_3_O_4_ or to the CuO/Co_3_O_4_ nanoparticles (Fig. 3C,D), the well attachment and ordered particle distribution was obtained. Further image analysis was obtained from the transmission electron microscopy (TEM) showing the formation of a spherical shape for the Co_3_O_4_ nanoparticles (Fig. 3E). In terms of the particle sizes obtained by the TEM, in CuO/Co_3_O_4_ composite, spherical shapes of Co_3_O_4_ with diameter 30 ± 5 nm and cubic shapes of CuO nanoparticles with diameter 40 ± 4.0 nm were observed, as depicted in Fig. 3F. Moreover, the characteristic bundles of CuO/Co_3_O_4_@MWCNTs (Fig. 3G) showed the diameter 12–23 nm for MWCNTs. Elemental identifications were approved by the energy dispersive X-ray analysis (EDX). Lastly, the EDX analysis for the chemical composition showed the contribution of Cu, Co, O, and C as the main elements, (Figure S1, supplementary materials).

Contact angle experiment was conducted in order to show the hydrophilic/hydrophobic characters of the coated surfaces with the nanocomposite, and its individual components. From Figure S2, supplementary materials, the values of measured contact angles of MWCNTs, CuO, Co_3_O_4_, CuO/Co_3_O_4_ and the CuO/Co_3_O_4_@MWCNTs were 145.9, 0.0, 62.8, 45.8 and 125.4. The values showed the hydrophilic character of CuO^59^ on the surface and less hydrophilic properties of Co_3_O_4_^60^, where MWCNTs^61^ give the lower hydrophilic properties which are correlated with the results that haven shown in the literature. Worth mentioning here that the water contact angles value of the Co_3_O_4_ was decreased after the addition of CuO forming the metal oxide nano-hybrid (CuO/Co_3_O_4_) from 62.8° to 45.8° due to the super hydrophilic of CuO, where the water contact angles of CuO/Co_3_O_4_@MWCNTs was also decreased from MWCNTs 145.9° to 125.4° due to the hydrophilic properties of the Co_3_O_4_.

### Electrochemical characterization of the CuO/Co_3_O_4_@MWCNTs modified SPEs

**Figure 4:**
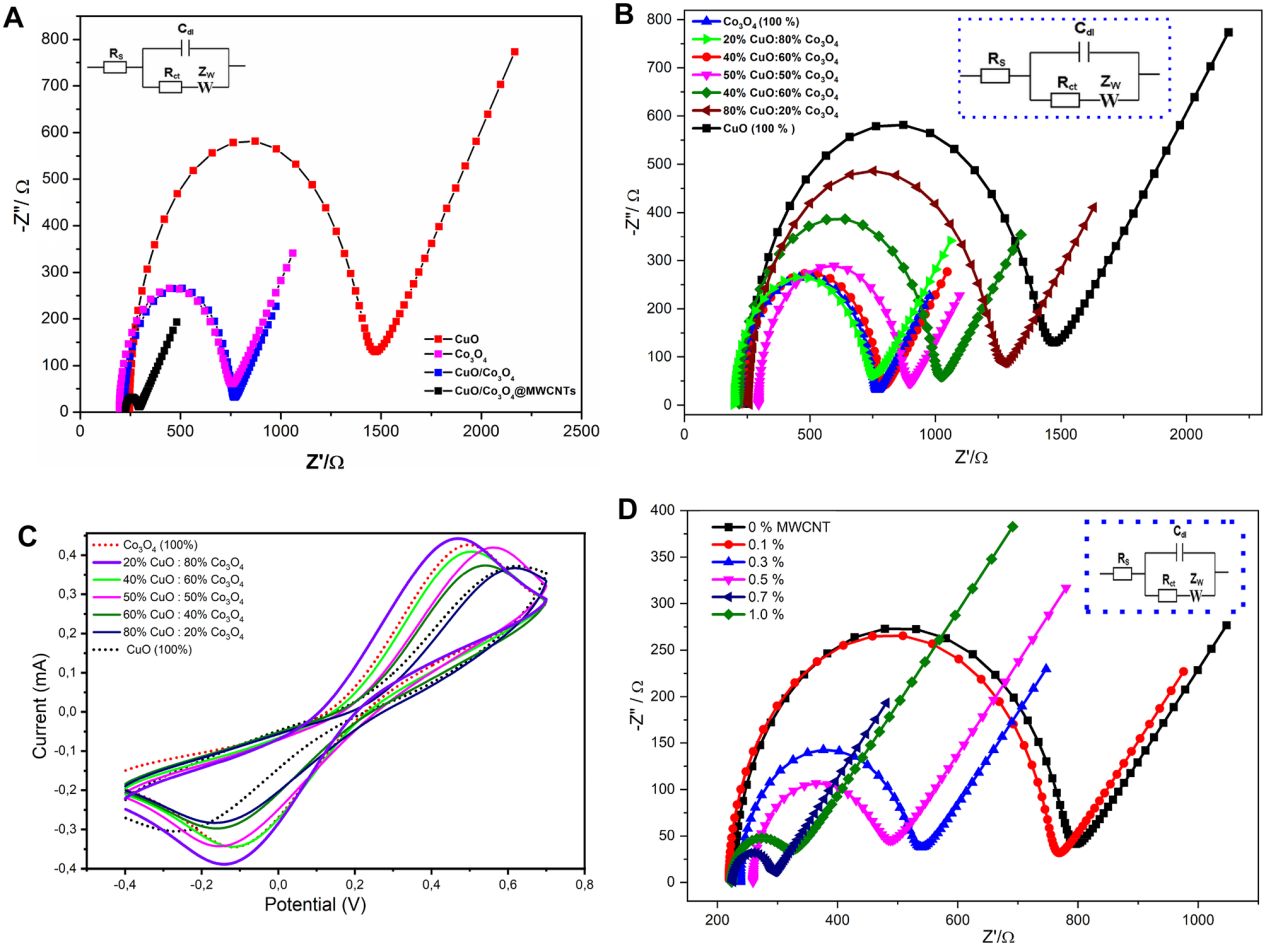

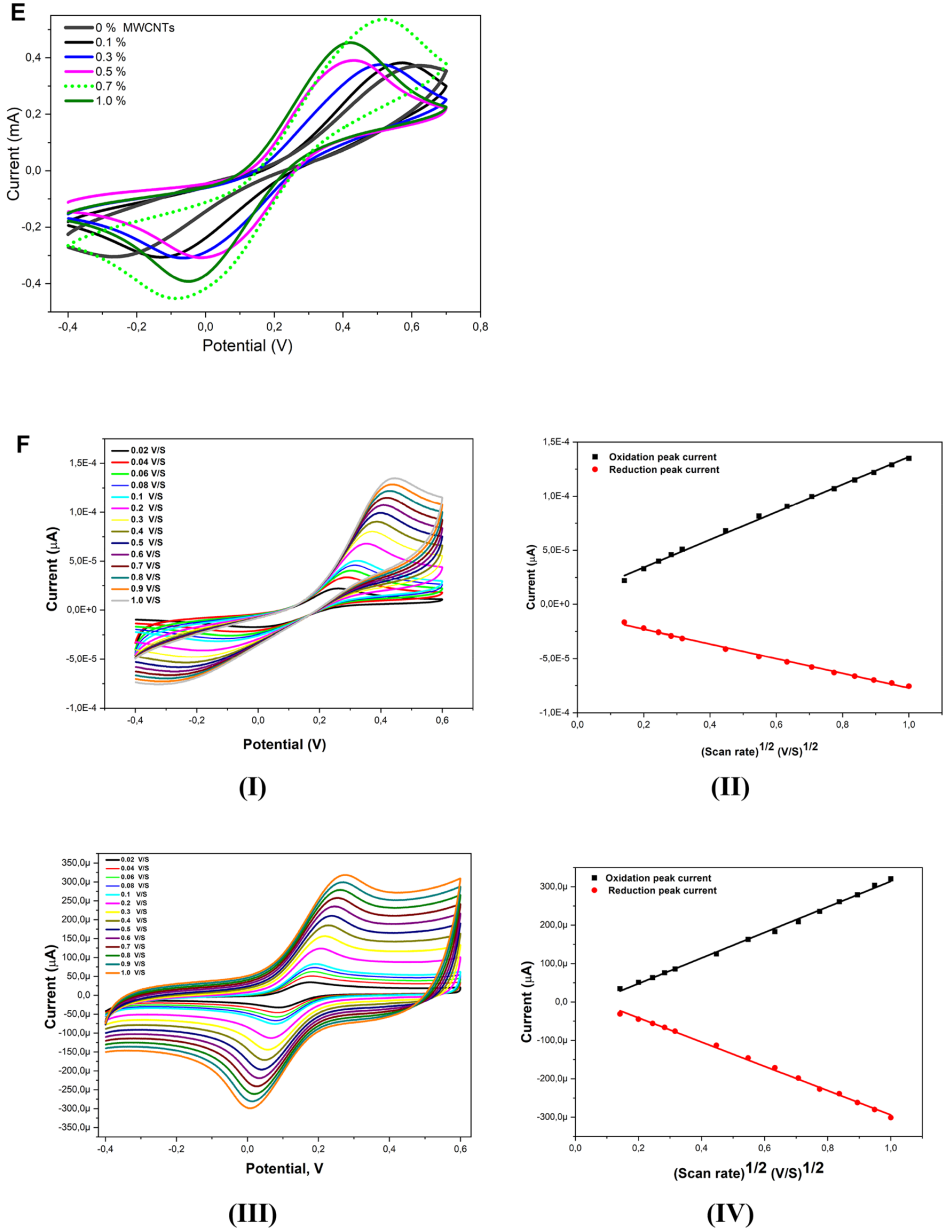
(**A**) Nyquist EIS spectra of CuO, Co_3_O_4_, CuO/Co_3_O_4_ and CuO/Co_3_O_4_@ MWCNTs modified SPEs using a solution containing 5.0 mM of redox probe ([Fe (CN)_6_]^3−/4−^) in 0.1 M KCl as the supporting electrolyte. (**B**) Nyquist EIS spectra different ratios of CuO/Co_3_O_4_ nanocomposite in CuO/Co_3_O_4_@MWCNTs modified SPEs using a solution containing 5.0 mM of the [Fe (CN)_6_]^3−/4−^ and 0.1 M KCl as the supporting electrolyte. (**C**) Cyclic voltammograms showing the effect of different ratios of the CuO/Co_3_O_4_ nanocomposite in CuO/Co_3_O_4_@MWCNTs modified SPEs using a solution of 5.0 mM of the [Fe (CN)_6_]^3−/4−^ and 0.1 M KCl as the supporting electrolyte. (**D**) Nyquist EIS spectra of different percentages of MWCNTs (0.1, 0.3, 0.5, 0.7 and 1%) in CuO/Co_3_O_4_@MWCNTs modified SPEs using a solution of 5.0 mM of the [Fe (CN)_6_]^3−/4−^ and 0.1 M KCl as the supporting electrolyte. (**E**) Cyclic voltammograms showing the effects of different percentages of MWCNTs (0.1, 0.3, 0.5, 0.7 and 1%) in CuO/Co_3_O_4_@MWCNTs modified SPEs using the solution of 5.0 mM of the [Fe (CN)_6_]^3−/4−^ and 0.1 M KCl as the supporting electrolyte. (**F**) Cyclic voltammograms and calibration curves showing the influence of scan rate changes (from 0.02 to 1.0 V/s) on the electrochemical performances of (I , II) unmodified and (III , IV) modified nanocomposite-based electrodes. The test has been conducted in the solution of 5.0 mM of the [Fe (CN)_6_]^3−/4−^ and 0.1 M KCl as the supporting electrolyte.

Electrochemical characteristics of the CuO, Co_3_O_4_, CuO/Co_3_O_4_ and the CuO/Co_3_O_4_@MWCNTs nanocomposites modified SPEs were firstly explored using the EIS in a solution containing 5.0 mM of Fe(CN)^3−/4−^ as the standard redox probe. As shown in Fig. 4A, distinguished impedimetric performances was provided by the different modified electrodes whereas a very high charge transfer resistance (R_ct_) with the value of 1150 Ω was obtained when the CuO modified-SPE was tested to reflect the semiconducting property of the copper oxide modified electrode. However, modified electrodes with the Co_3_O_4_ exhibited a lower charge transfer resistance (532 Ω) than the CuO-based electrode. Consequently, a dramatic drop in the value of the R_ct_ was attained when the carbon nanotubes was added to the nano-hybrid of the metal oxides that led to a very low resistance value of 61 Ω obtained from the whole nanocomposite (CuO/Co_3_O_4_@MWCNTs).

Moreover, the EIS responses of modified electrodes with different percentages of CuO to Co_3_O_4_ (0:100, 20:80, 40:60, 50:50, 60:40, 80:20 and 100: 0%) were obtained whereas the lowest charge transfer resistance was obtained when the percentage ratio of CuO to Co_3_O_4_ was 20:80. Increasing the concentration of CuO led to increasing the R_ct_ values, as shown in Fig. 4B and Table S2. In parallel to the EIS measurements, changes in the voltammetric behaviors represented by the oxidation/reduction peak currents of the redox species at the modified electrode surfaces were monitored at different percentages of CuO to the percentages of Co_3_O_4_. As shown in Fig. 4C and Table S2, the SPE modified with different ratios exhibited higher redox signal than the modified with CuO alone. However, the highest voltammetric signal was achieved when the ratio of 20:80 was used for the electrode modification.

Additionally, the effect of mixing the metal oxide nano-hybrid with the carbon nanotubes was studied using the CV and EIS at different concentrations (0, 0.1, 0.3, 0.5, 0.7, and 1.0%) of the MWCNTs, as depicted in Fig. 4D,E. The overall electrochemical signals, either the voltammetric or the impedimetric responses, were improved after the addition of the carbon nanotubes. This was very clear since the charge R_ct_ values significantly decreased, while the oxidation/reduction voltammetric currents were much improved (see table S3). Accordingly, the final selected composition of the nanocomposite was 19.3: 80: 0.7%, for the CuO, Co_3_O_4_, and MWCNTs, respectively.

For further electrochemical characterizations, the effective electrochemically active surface area (i.e. the conductive surface area) of the unmodified and modified electrodes with the nanomaterials (unmodified, the CuO, CO_3_O_4_, CuO/Co_3_O_4_, and CuO/Co_3_O_4_@MWCNTs modified SPEs) were evaluated using the scan rate effect. Accordingly, the electrochemically active surface area of each electrode was calculated by the Randles–Sevcik Eq. (2):

2 $$ I_{p} = 2.69*10^{5} *{\text{n}}^{3/2} *{\text{A}}*{\text{D}}^{1/2} *C\mu^{1/2} $$

where I_p_ is the voltammetric peak current, n is the number of electron transfer, A is the electrochemical active area (cm^2^), D is the diffusion coefficient (cm^2^/s), C is the concentration of the electrochemical redox species [Fe(CN)_6_]^3−/4−^ (mol/l), and the *μ* is the scan rate (V/s). From the slopes of the *I*_*p*_ versus the square root of the scan rate (*μ*^1/2^), the calculated electroactive surface areas of the unmodified, or the modified electrodes with the CuO, Co_3_O_4_, CuO/Co_3_O_4_ and CuO/Co_3_O_4_@MWCNTs were found to be 0.044, 0.0388, 0.0723, 0.0749 and 0.1148 cm^2^, respectively. These results indicated that the modified screen-printed electrodes with the nanocomposite provided the largest expanded electrochemically active surface area. Eventually, the modified electrodes with the nanocomposite exhibited higher reversibility and diffusion controlled voltammetric process (As shown in 4F (III, IV)), while the unmodified electrodes showed an irreversible and adsorption controlled voltammetric process (As depicted in Fig. 4F (I, II)).

### Electrochemical sensing of urea using CuO/Co_3_O_4_@MWCNTs nanostructure

**Figure 5:**
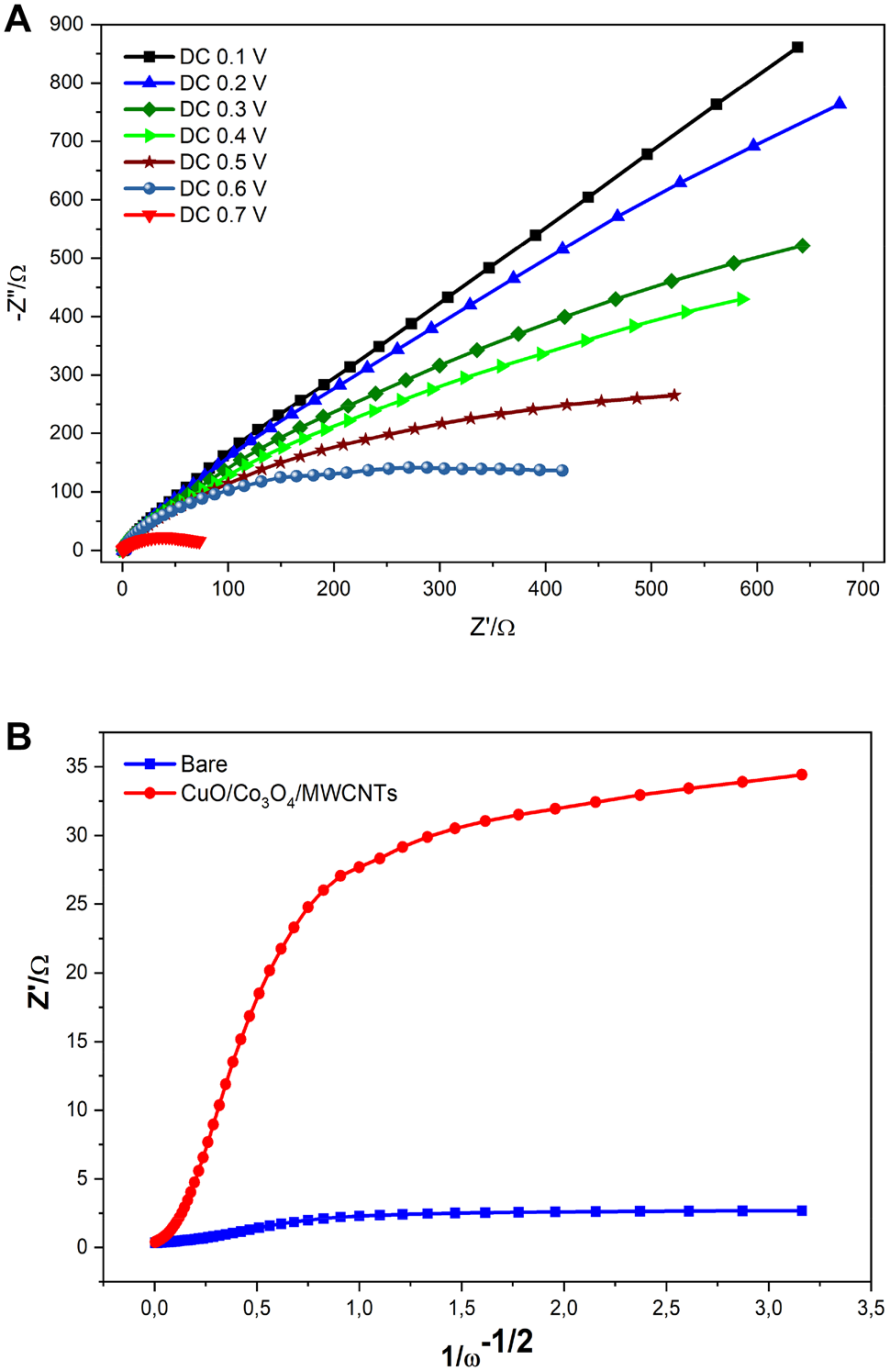
(**A**) Nyquist EIS spectra showing the applied direct current potential (DC voltages) on the impedimetric performance of the nanocomposite-based electrodes. The experiments have been conducted in KOH solution (0.1 M) over the applied frequency range from 0.1 to 10^5^ Hz and alternating current (AC) amplitude of 5.0 mV sinusoidal modulation. (**B**) Coverage rate determination for the unmodified and nanocomposite-based electrodes. The test has been performed in KOH solution (0.1 M).

To improve the response towards electrochemical detection, the newly characterized and selected nanocomposite (CuO/Co_3_O_4_@MWCNTs) has been tested for the direct, and label free impedimetric sensing of urea in real samples. Therefore, for the optimization assay, effective parameters were studied. Firstly, the influence of the direct current (DC) on the impedimetric signals was tested by changing the polarization potential from 0.0 to 0.7 V, while the EIS signal was monitored at every direct current (DC) value (as demonstrated in Fig. 5A and Table S3). The decrease in the values of charge transfer resistance (R_ct_) and Warburg resistance (Z_W_) were strongly dependent on the applied DC values. The lowest resistance values were reached when the DC of 0.7 was applied to be chosen for the further optimizations.

Secondly, for electrode coverage rate determination, applying the same experimental conditions for EIS measurements of the CuO/Co_3_O_4_@MWCNTs/SPE (0.7 V of polarization, a frequency range between 0.1 to 10^5^ Hz, and with amplitude of 5 mV), the impedance behavior before and after modification with the nanomaterials were recorded. Accordingly, the coverage rate (Ф) was calculated from the plot of real impedance part (Y-axis) before and after the nanomaterial modification vs the inverse of the square root of the sinusoidal excitation pulsation (ω^−1/2^) (As shown in Fig. 5B). In the low frequency region, the linear range intercept is at ω^−1/2^ with the real impedance axis (ordinary axis) on ionic charge transfer resistance (R_ct_). The calculated coverage rate from Eq. (3) was obtained to about 92%

3 $$ \Phi = 1 - \left[ {{\text{R}}_{{{\text{ct}}}} \left( {{\text{unmodified}}\;{\text{SPE}}} \right){\text{/R}}_{{{\text{ct}}}}^{\prime } \, \left( {{\text{CuO/Co}}_{3} {\text{O}}_{4} @{\text{MWCNT/SPE}}} \right)} \right] $$

Ф is the coverage rate, R_ct_ is the ionic charge transfer of the unmodified electrode, and ${\text{R}}_{{{\text{ct}}}}^{\prime }$ is the ionic charge transfer of the nanocomposite-based electrode.

To this end, the sensor surface that is fully covered with the nanomaterials could be exploited for the catalytic oxidation of urea at its surface providing sensitive changes in the electrochemical signals which are correlated with the added urea concentrations. Accordingly, the expected surface reaction mechanism behind the redox reaction of urea could be illustrated as follows:

$$ {\text{Co}}\left( {{\text{NH}}_{2} } \right)_{2} + 6{\text{OH}}^{ - } > {\text{N}}_{2} + {\text{CO}}_{2} + {\text{H}}_{2} {\text{O}} + 6{\text{e}}^{ - } $$

### Standard calibration curves of urea

**Figure 6:**
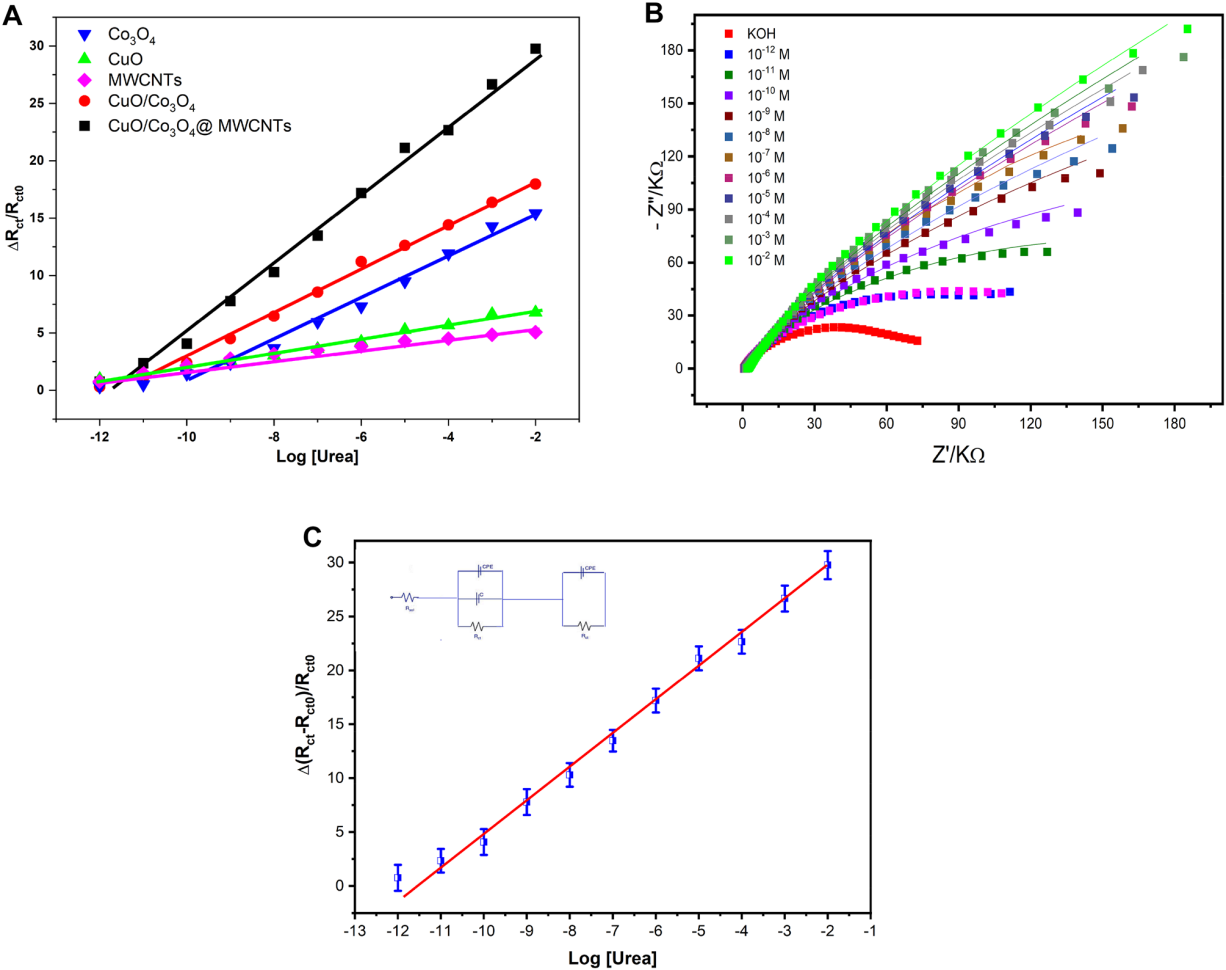
(**A**) Sensing performance testing towards different concentrations of the targeting anaylte (Urea) using different modified electrode surfaces. (**B**) Standard impedimetric calibration curve at the optimized conditions. Urea concentrations were ranged from 1.0 × 10^−12^ to 1.0 × 10^−2^ M. The impedance spectra were recorded at polarization potential of 700 mV in solution, frequency range of 0.1 to 10^5^ Hz with an amplitude of 5.0 mV sinusoidal modulation. (**C**) Linearity range extracted from the standard calibration plot using a designed equivalent circuit (inset image).

At the optimum EIS conditions, the performance of unmodified and modified electrodes with individual nanomaterials (MWCNTs, CuO, Co_3_O_4_, CuO/Co_3_O_4_ and CuO/Co_3_O_4_@ MWCNTs) towards the direct detection of different concentrations of urea were evaluated. As shown in Fig. 6A, various concentrations of urea were injected into the electrochemical cell while the EIS measurements were collected and the Nyquist plots of impedance spectra were evaluated, while the changes in the charge transfer resistances (∆R_ct_) were presented. As a result, a limited linear range of 10^−10^–10^−2^ M was obtained from the electrodes modified with MWCNTs, CuO, Co_3_O_4_ and CuO/Co_3_O_4_. Accordingly, the limited sensitivity of each of these modified electrodes was calculated and found to be 0.41, 0.61, 1.63 and 1.87 for the MWCNTs, CuO, Co_3_O_4_ and CuO/Co_3_O_4_, respectively. Interestingly, the Nyquist plots of impedance spectra of modified electrodes with the CuO/Co_3_O_4_@MWCNTs (see Fig. 6B) exhibited the highest sensitivity (3.01 ± 0.085) and the widest linear range (10^−12^ to 10^−2^ M) with the regression coefficient R^2^ = 0.9961. Therefore, the limit of detection was calculated and found to be 2.23 × 10^−13^ M, as shown in Fig. 6C. For the quantitative data analysis, an equivalent circuit was designed and the fitting results were tabulated in Table 1 showing the extracted values for the (R_s_, R_ct_ and the CPE) obtained from each concentration of urea. Thus, the acquired synergetic features of the nanocomposite-based electrodes were exploited for the direct and high sensitivity quantification of urea solutions.

**Table 1:** Fitting data for CuO/Co_3_O_4_ @ MWCNTs modified based impedimetric SPE sensor for different urea concentrations.

Urea conc. M	*R*_s_ (Ω)	CPE (µF)	*C* (µF)	*R*_ct1_ (KΩ)	CPE (µF)	*R*_ct2_ (KΩ)
0	362.6	6.85	1.441	83.27	39.02	3.364
10^−12^	533.2	6.692	1.386	167.1	33.61	25.41
10^−11^	615.7	6.59	1.312	278.55	30.22	56.32
10^−10^	685.1	6.49	1.199	422.1	29.56	92.35
10^−9^	750.4	6.09	1.089	730.9	28.56	164.9
10^−8^	789	6.02	1.068	940.8	27.09	220.6
10^−7^	989.2	5.96	1.002	1205	26.87	341.7
10^−6^	1012	5.74	0.9522	1515	25.94	404.3
10^−5^	1066	5.65	0.8674	1842	24.71	437.4
10^−4^	1356	5.45	0.7479	1970	23.87	468.9
10^−3^	1534	4.37	0.5509	2303	23.08	636.1
10^−2^	2025	4.04	0.548	2561	19.78	719.6

A comparison between the analytical performance of the prepared sensor and other reports for urea sensors was presented in Table 2. The newly developed non-enzymatic biosensors are providing a wide linear range from 10^−12^ to 10^−2^ M , and a very high sensitivity with the lower detection limit of 2.23 × 10^−13^ M which is lower than the other sensors in the literature^62–82^.

**Table 2:** Comparison of analytical performance of other reported urea sensors with the proposed sensor.

Electrodes	Enzymatic/non enzymatic	L.R	L.D.L	Sensitivity	Response time (s)	Ref
Urease/ZnO-chitosan composite/ITO	Enzymatic	0.8–16.6 mM	499 μM	0.13 μA mM^−1^ cm^−2^	10	^62^
Urease/chitosan-Fe_3_O_4_ composite/ITO	Enzymatic	0.8–16.6 mM	333 μM	12.50 μA mM^−1^ cm^−2^	10	^63^
Urease/ZrO_2_ thin film/Au	Enzymatic	0.8–16.6 mM	80 μM	0.07 μA mM^−1^ cm^−2^	10	^5^
Urease/poly(glycidyl methacrylate-covinylferrocene/GCE	Enzymatic	0.1–1.5 mM	60 μM	0.32 nA mM^−1^	3	^64^
Urease/ZnO-MWCNT/ITO	Enzymatic	1.6–16.6 mM	238 μM	43.02 μA mM^−1^ cm^−2^	4	^65^
Urease/nanoporoussili calite particles/Au	Enzymatic	0.05–15 mM	20 μM	–		^66^
NF/urease/Yb_2_O_3_/GCE	Enzymatic	0.05–19 mM	2 μM	124.84 μA mM^−1^ cm^−2^	3	^67^
Sulfonated graphene/polyaniline nanocomposite	Enzymatic	0.12–12.3 mM	0.050 mM	0.85 μA mM^−1^ cm^−2^	5	^68^
Polyaniline-Nafion	Enzymatic	1.0 íM to 10 mM	0.5 íM	0.769 μA mM^−1^ cm^−2^	40 s	^69^
Urease/chitosan/cobalt oxide (CS/Co_3_O_4_) nanocomposite	Enzymatic	1. 9 10^−4^ and 8. 9 10^−2^ M	–	45 mV	12 s	^70^
Urease/polyvinyl alcohol (PVA) and polyacrylamide (PAA) composite polymer	Enzymatic	1–1000 mM	–	–	120 s	^71^
Urease/polyaniline grafted conducting hydrogel	Enzymatic	1.5–1000 mM	60 nM	878 μA mM^−1^ cm^−2^		^72^
A porous silk fibroin membrane with immobilized urease was mounted in a polydimethylsiloxane (PDMS) sensor	Enzymatic	0.1–20 mM	–	–		^73^
Zinc oxide nanorods (ZnO NRs) on Ag sputtered glass substrate	Enzymatic	0.001–24.0 mM	10 μM	41.64 μA/mM cm^2^		^74^
Nonactine (PVC-membrane electrode)	Enzymatic	1 × 10^−5^ to 1 × 10^−2^ M	–	40.3 mV	1–2 min	^75^
Urease-immobilized PPy film electrode	Enzymatic	0.5 to 10 mM	–	43.4 mV/p Urea		^76^
Graphene-PANi/GCE	Non-enzymatic	10–200 μM	5.88 μM	226.9 μA μM^−1^ cm^−2^	–	^77^
Ni-MOF/MWCNT	Non-enzymatic	0.01–1.12 μM	3 μM	685.16 μA μM^−1^ cm^−2^	< 10	^78^
Ag-ZnO/GCE	Non-enzymatic	26.3–427 μM	13.98 μM	0.1622 μA μM^−1^ cm^−2^	–	^79^
NF/Ag–N-SWCNTs/GCE	Non-enzymatic	66 nM–20.6 mM	4.7 nM	141.44 μA mM^−1^ cm^−2^	3	^80^
MIP TiO_2_ thin film	Non-enzymatic	0.04–120 μM	0.01 μM	–	–	^81^
NiCoO2 nanoneedles	Non-enzymatic	0.01–5 mM	1.0 mM	–	–	^82^
CuO/Co_3_O_4_@MWCNTs	Non-enzymatic	10^−12^–10^−2^ M (R^2^ = 0.996)	0.7 PM	4.64 (R_ct_ − R_ct0_)/R_ct0_)	–	Recent work

### Sensors selectivity testing

**Figure 7:**
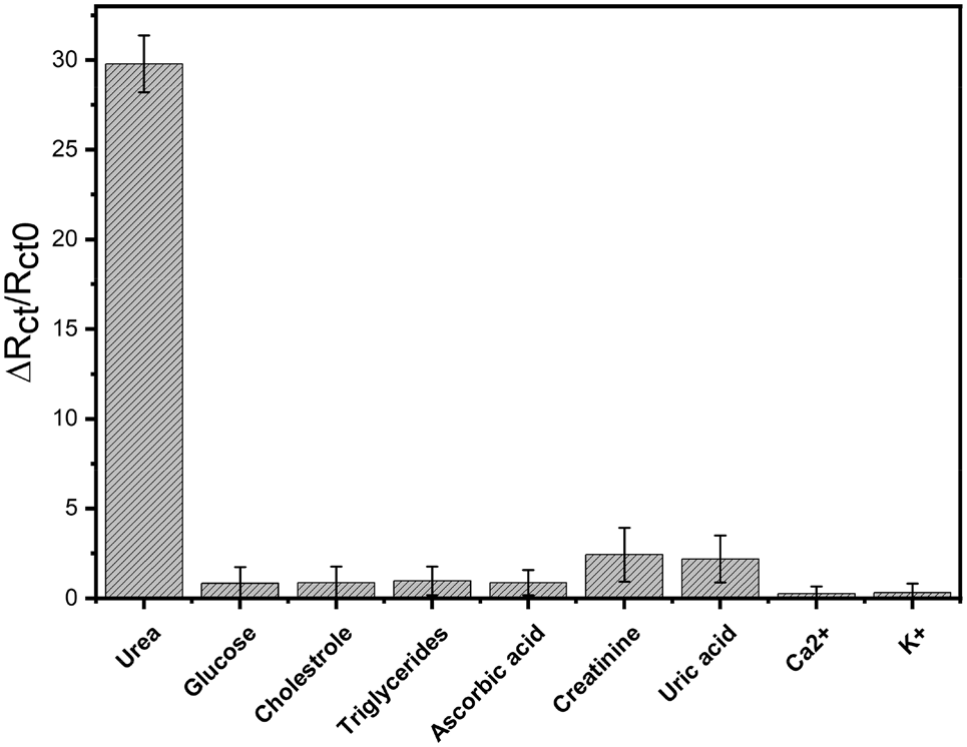
Interference study showing the cross-reactivity influence of non-targeting molecules on the impedimetric responses of the proposed urea-sensor. A single concentration (10 mM) was tested for each of the foreigner molecule. All optimized conditions have been applied.

For testing the selectivity of the proposed non-enzymatic biosensor, EIS responses of the nanocomposite-based electrode were individually evaluated against several non-targeting species (foreigner targets) including glucose, cholesterol, triglycerides, ascorbic acid, creatinine, uric acid, Ca^2+^ and K^+^. Meanwhile, the EIS response towards urea was included as a positive control, Fig. 7. The results confirmed that the presence of non-targeting/interfering molecules did not cause any significant changes in the generated EIS responses, while urea was the only reason behind the reasonable impedimetric signals. Accordingly, the newly proposed non-enzymatic biosensors provided high sensitivity and high selectivity for urea detection.

### Repeatability, reproducibility, and lifetime of the non-enzymatic biosensor

Repeatability, reproducibility and the lifetime of the nanocomposite-based electrode were studied. Firstly the repeatability feature was tested by taking several-measurements (n = 6) with a fixed concentration of urea (1 µM) for one electrode. The charge transfer resistance (∆ R_ct_/R_ct0_) of each experiment was calculated and compared to each other. The results shown in Figure S3A (Supplementary materials) were very close and no significant difference was obtained.

For the reproducibility test, multiple fabricated electrodes (five electrodes) were used for the direct detection of a single urea concentration (1 µM), while the obtained charge transfer resistance (∆R_ct_/R_ct0_) for each electrode was calculated and presented in Figure S3B, supplementary materials. As a result, good repeatability and reproducibility results were confirmed with relative standard deviation (RSD) of 1.26, and 1.03%, respectively.

Eventually, the lifetime of one single fabricated electrode was determined over four weeks of continuous tracking. High stability was obtained, as shown in Figure S3C supplementary materials. The main advantage of using nanocomposite-based electrodes is to avoid the use of enzymes of mediated reagents which might affect the sensor’s stability and lifetime.

### Determination of urea in real samples

Spiked tap water and cow milk samples with certain standard urea concentrations were prepared and tested using the newly fabricated electrode. As shown in Table 3, high recovery rates (95–104%) were obtained from all the artificially urea contaminated samples. Thus, the sensor could be used for any other real environmental or biological samples.

**Table 3:** Determination of urea in real samples.

Sample no	Urea concentration measurement by impedimetric sensor (mg/dl)	Recovery%
Milk		
Sample 1	35.2	–
Sample 1 + 20 mg/dl urea added	56.1	104.5
Sample 1 + 40 mg/dl urea added	77.5	105.5
Sample 2	25.5	–
Sample 2 + 20 mg/dl urea added	46.8	106.5
Sample 2 + 40 mg/dl urea added	65.1	99.0
Sample 3	27.8	–
Sample 3 + 20 mg/dl urea added	48.1	101.5
Sample 3 + 30 mg/dl urea added	58.1	101.0
Water		
Sample 1	30.2	–
Sample 1 + 20 mg/dl urea added	49.9	98.5
Sample 2	28.6	–
Sample 2 + 30 mg/dl urea added	59.1	101.6
Sample 3	31.5	–
Sample 3 + 40 mg/dl urea added	71.6	100.2

## Conclusion

Exploring a new class of hybrid metal oxide nanostructure could offer unique advantages that can be utilized to enhance the poor electrical conductivity and weak charge transfer processes. In this study, synthesis and characterization of a new class of metal oxides nanostructure (CuO/Co_3_O_4_) and their integration with the MWCNTs to produce a uniformed nanocomposite (CuO/Co_3_O_4_@MWCNTs) were successfully performed. The explored synergetic electro-catalytic features acquired by the newly obtained nanocomposites were exploited for the construction of a direct non-enzymatic biosensing platform to be exploited for the impedimetric detection of urea in real samples. A full impedimetric assay was optimized and all controlling factors were identified. Thus, disposable screen-printed electrodes modified with the nanocomposite exhibited a very high sensitivity with a wide linear range from 10^−12^ M to 10^−2^ M, and the limit of detection of 0.223 pM. In addition, a high selectivity was also obtained when several non-targeting molecules were exposed to the sensor. Accordingly, the sensor was applied for the fast and direct urea determination in water and milk samples. With high stability, reproducibility and direct application, the newly designed sensor is very promising for the quality assurance of food and environmental samples that might contain urea in their matrices.

## Supplementary Information

Supplementary Information.

## Data Availability

All data generated or analyzed during this study are included in this published article (and its Supplementary Information files).
