# Supplementary material for: Non-enzymatic disposable electrochemical sensors based on CuO/Co3O4@MWCNTs nanocomposite modified screen-printed electrode for the direct determination of urea
Source: Sci Rep. 2023 Feb 4;13:2034. doi: 10.1038/s41598-023-28930-4 (PMC9899286; doi:10.1038/s41598-023-28930-4)
Supplement: Supplementary file 1 — Supplementary Information. [file 41598_2023_28930_MOESM1_ESM.doc]

## Non-enzymatic disposable electrochemical sensors based on CuO/Co_3_O_4_@MWCNTs nanocomposite modified screen-printed electrode for the direct determination of urea

**Hend S. Magar^a*^, Rabeay Y. A. Hassan^b^, Mohammed Nooredeen Abbas**^a^

^a^Applied Organic Chemistry Department, National Research Centre Dokki, P.O. Box.12622, Cairo, Egypt,

^b^Nanoscience Program, University of Science and Technology (UST), Zewail City of Science and Technology, Giza 12578, Egypt

**^*^Corresponding author**

Hend S. Magar, PhD

National Research Centre (NRC)

Email address: hs.ameen@nrc.sci.eg

Phone: +201121926682


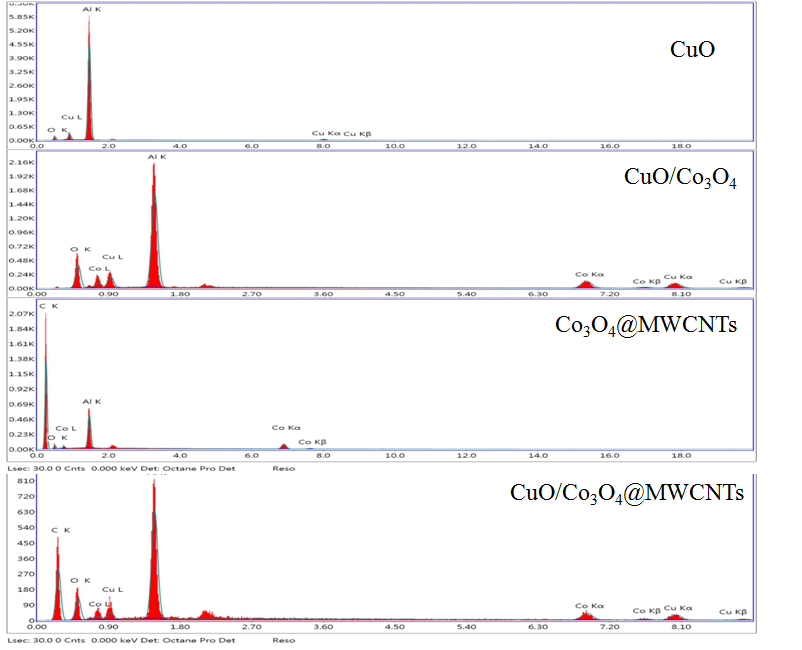


**Figure S1**. EDX analyses images of the CuO , CuO/ Co_3_O_4_ , C) Co_3_O_4_ @ MWCNTs and CuO/ Co_3_O_4_ @ MWCNTs modified Aluminum sheet.

| 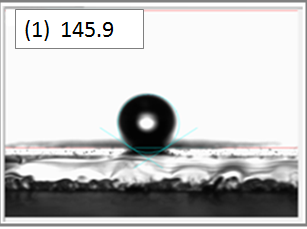 | 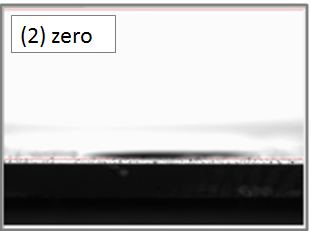 | 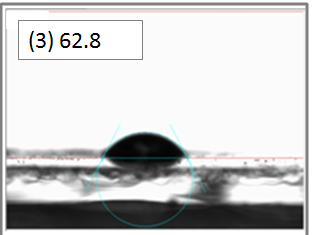 |
| --- | --- | --- |
| 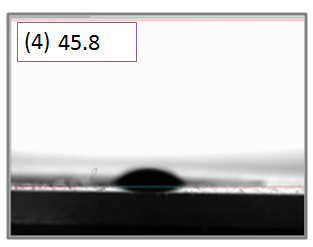 | 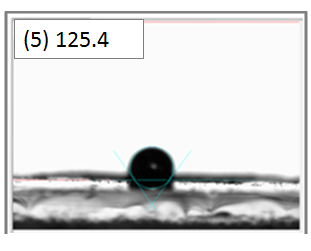 |  |

**Figure S2**. Contact angle images of (1) MWCNTs, (2) CuO , (3) Co_3_O_4_ , (4) CuO/ Co_3_O_4_ (600^o^C) and (5) CuO/ Co_3_O_4_@ MWCNTs .


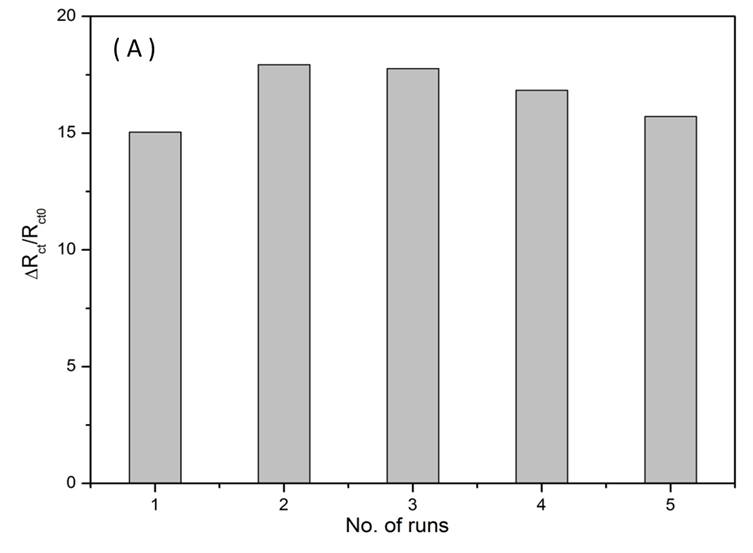


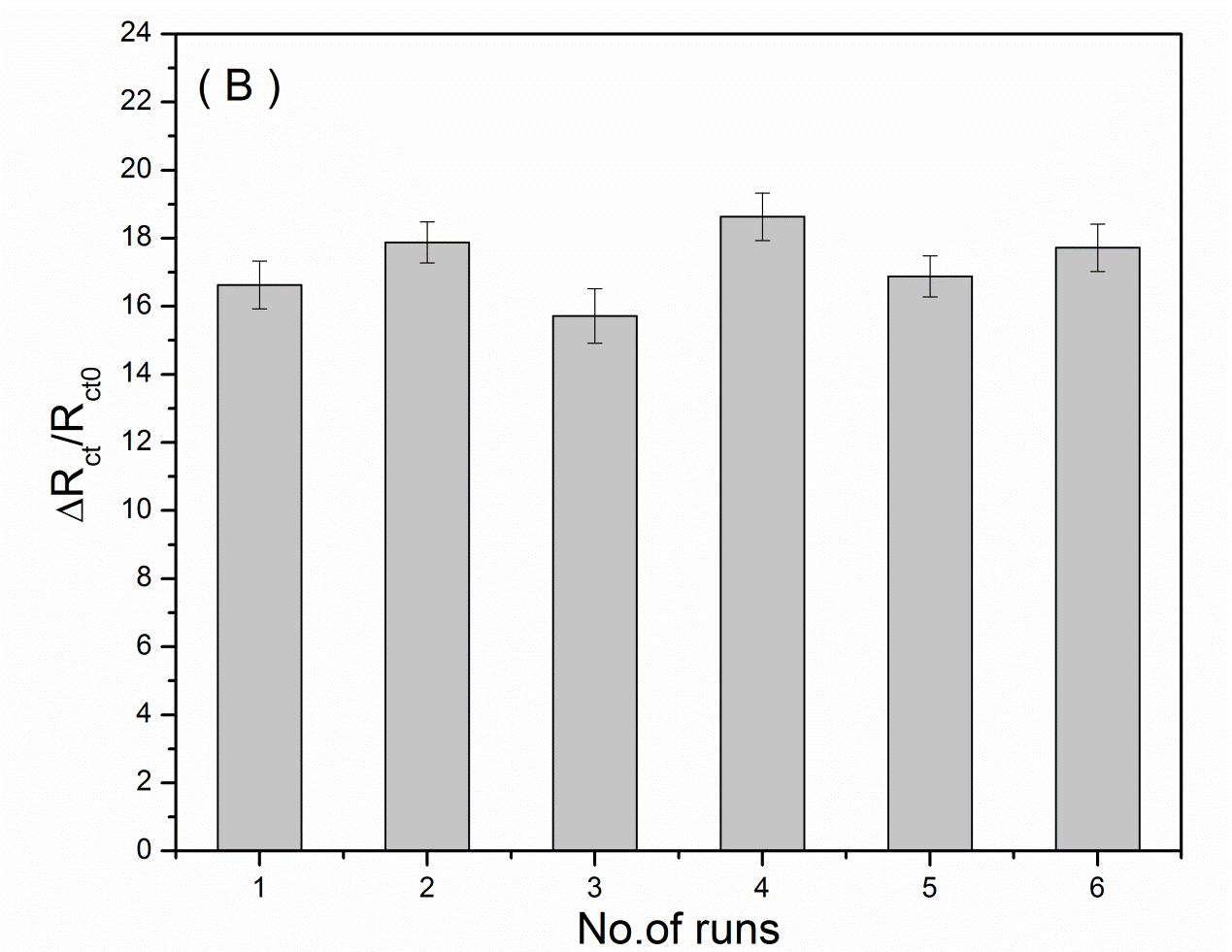


**
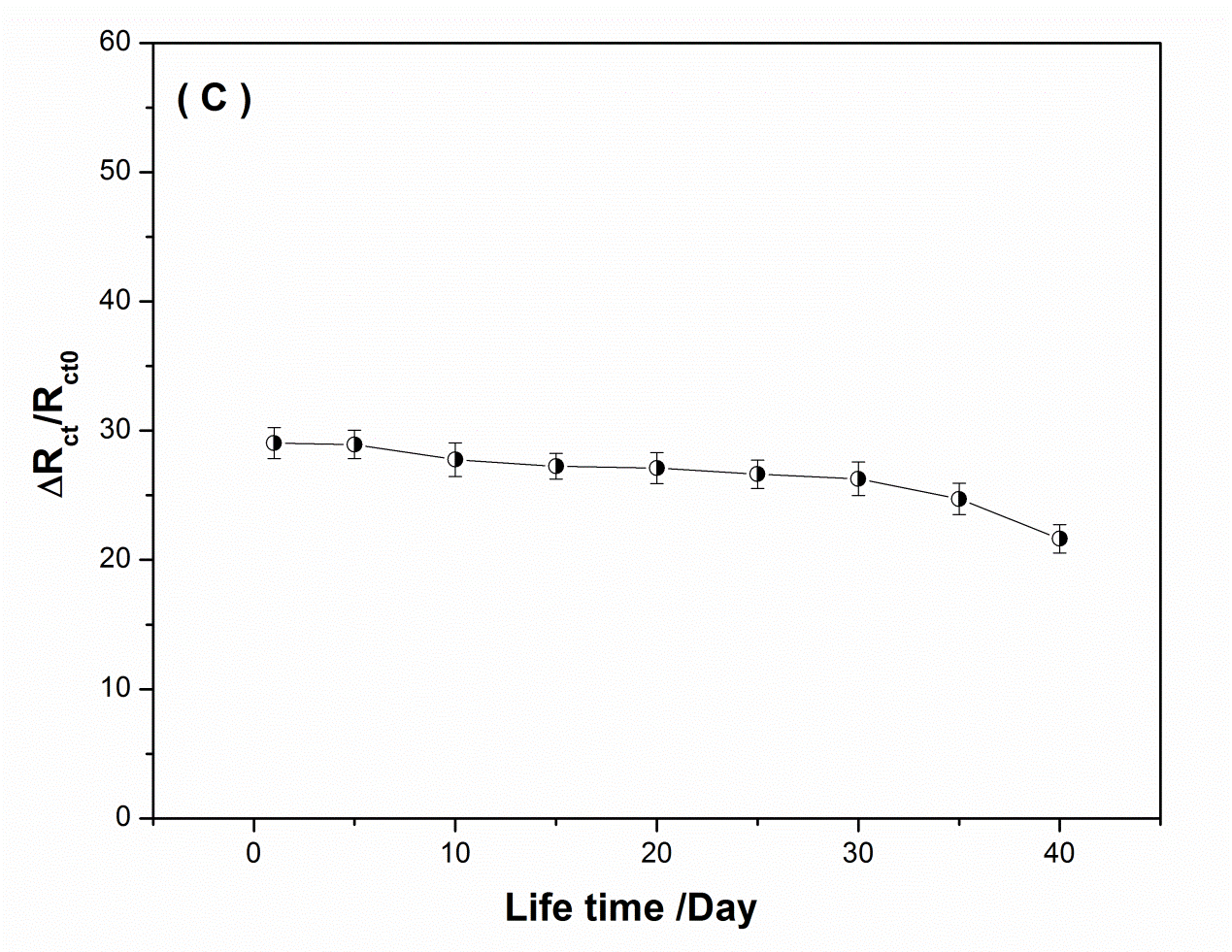
**

**Figure S3**. (A) Repeatability, (B) reproducibility of the CuO/ Co_3_O_4_ @MWCNTs SPE in a solution of 1µM of urea in KOH , 0.1 M ( at 0.7 V). **(C)**  Life time of the proposed sensor response toward 10 mM concentration of urea in a solution of KOH , 0.1 M ( at 0.7 V).

**Table S1:** Calculated crystalline size and dislocation density of the presented nanomaterials

| **Sample** | 2θ | FWHM ( ^o^) | **Crystal size**  **(D, nm)** | **σ** |
| --- | --- | --- | --- | --- |
| **MWCNTs** | 25.4 | 5.068 | 0.0283 | 70.4 |
| **CuO** | 38.8 | 0.357 | 0.344 | 5.8 |
| **Co_3_O_4_** | 36.9 | 0.212 | 0.63 | 3.13 |
| **CuO/Co_3_O_4_** | 35.1 | 0.343 | 0.11 | 16.7 |
| **Co_3_O_4_@ MWCNTs** | 36.5 | 0.33 | 0.36 | 5.5 |
| **CuO/Co_3_O_4_@MWCNTs** | 38.8 | 1.20 | 0.104 | 19.5 |

**Table S2 :**  CV ( I_a_,  I_c_,  E_oxd_.,  E_red_., ΔE) & EIS parameters of different ratios of CuO/ Co_3_O_4_ in CuO/Co_3_O_4_@MWCNTs modified SPE using a solution of 5 mM [Fe (CN) _6]_^3-/4-^ and 0.1M KCl as supporting electrolyte .

| **Electrode type**  CuO :Co_3_O_4_ | ***I_a_***  **(10^-4^ A)** | ***I_c_***  **(10^-4^ A)** | **E *_oxd_*.**  **(V)** | **E*_red._***  **(V)** | **E_1/2_**  **(V)** | **R_s_**  **(Ω)** | **R_ct_**  **(Ω)** | **C**  **(µF)** | **W / 1e-3**  **(Ω)** |
| --- | --- | --- | --- | --- | --- | --- | --- | --- | --- |
| 0:100 | -1.94 | 1.95 | 0.497 | -0.112 | 0.192 | 220.3 | 532.5 | 0.897 | 3.33 |
| 20 : 80 | -1.95 | 2.08 | 0.472 | -0.134 | 0.169 | 195.7 | 524.3 | 0.957 | 3.12 |
| 40 : 60 | -1.52 | 1.60 | 0.504 | -0.113 | 0.195 | 219.4 | 543.5 | 0.797 | 3.22 |
| 50 : 50 | -1.45 | 1.73 | 0.560 | -0.151 | 0.204 | 295.3 | 573.5 | 0.589 | 3.93 |
| 60 : 40 | -1.29 | 1.40 | 0.542 | -0.159 | 0.191 | 218.5 | 769.7 | 0.484 | 3.92 |
| 80 : 20 | -0.97 | 1.09 | 0.603 | -0.162 | 0.220 | 258.5 | 962.9 | 0.397 | 4.10 |
| 100 : 0 | -1.31 | 0.941 | 0.615 | -0.261 | 0.177 | 246.0 | 1150 | 0.579 | 5.11 |

**Table S3:**  CV ( I_a_,  I_c_,  E_oxd_.,  E_red_., ΔE) & EIS parameters of *CuO/ Co_3_O_4_ @ MWCNTs* modified SPE containing different ratios of MWCNT ( 0.1 , 0.3 , 0.5, 0.7 and 1 % ) in a solution of 5 mM [Fe (CN) _6]_^3-/4-^ and 0.1M KCl

| **Electrode type**  **MWCNT %: CuO % :** Co_3_O_4_**%** | ***I_a_***  **(10^-4^ A)** | ***I_c_***  **(10^-4^ A)** | **E *_oxd_*.**  **(V)** | **E*_red._***  **(V)** | **E_1/2_**  **(V)** | **R_s_**  **(Ω)** | **R_ct_**  **(Ω)** | **C**  **(µF)** | **W**  **(KΩ)** |
| --- | --- | --- | --- | --- | --- | --- | --- | --- | --- |
| 0 : 20 : 80 | -1.22 | 1.495 | 0.57 | -0.125 | 0.22 | 220.3 | 532.5 | 0.897 | 3.33 |
| 0.1 : 20 : 80 | - 1.85 | 1.895 | 0.57 | -0.127 | 0.221 | 210.8 | 523.0 | 1.06 | 3.28 |
| 0.3 : 20 : 80 | -1.95 | 2.152 | 0.512 | -0.06 | 0.226 | 237 | 280.4 | 2.61 | 2.99 |
| 0.5 : 20 : 80 | -2.23 | 2.69 | 0.43 | -0.01 | 0.21 | 259 | 205.4 | 3.728 | 2.82 |
| 0.7 : 20 : 80 | -3.18 | 3.45 | 0.529 | -0.082 | 0.223 | 226 | 61.83 | 4.96 | 1.6 |
| 1 : 20 : 80 | -2.77 | 3.02 | 0.429 | -0.052 | 0.188 | 173 | 86.65 | 4.12 | 2.3 |

**Table S4:   EIS parameters of *CuO/Co_3_O_3_@MWCNTs*/SPE structure for different polarizations from 0.1 – 0.7 V (frequency range from 0.1 to 10^5^ Hz and amplitude of 5.0 mV sinusoidal modulation in KOH 0.1M solution.**

| **Potential**  **V** | ***R*_s_**  **(Ω)** | **CPE**  **(µF)** | ***C***  **(µF)** | ***R*_ct1_**  **(KΩ)** | **CPE**  **(µF)** | ***R*_ct2_**  **(KΩ)** |
| --- | --- | --- | --- | --- | --- | --- |
|  |  |  |  |  |
| 0.1 | 955.5 | 1.018 | 0.102 | 9.837e8 | 3.25 | 100.1 |
| 0.2 | 848 | 1.21 | 0.1135 | 8.63e8 | 3.98 | 94.5 |
| 0.3 | 760.9 | 1.299 | 0.139 | 5.601e6 | 5.14 | 74.9 |
| 0.4 | 919.8 | 1.312 | 0.1428 | 2.48e5 | 8.63 | 23.19 |
| 0.5 | 673.2 | 1.622 | 0.202 | 11880 | 16.21 | 16.77 |
| 0.6 | 670 | 2.034 | 0.673 | 603.5 | 18.45 | 11.70 |
| 0.7 | 449.4 | 4.952 | 0.951 | 86.11 | 35.36 | 5.606 |
